# Daytime eccentric exercise and its impact on inflammatory markers and antioxidant defenses in physically active young men

**DOI:** 10.3389/fimmu.2025.1655034

**Published:** 2025-09-17

**Authors:** Baikui Xu, Shuai Guo

**Affiliations:** ^1^ School of Physical Education, Shandong Women’s University, Jinan, Shandong, China; ^2^ Department of Basic Sciences, Shandong Xiehe University, Jinan, Shandong, China

**Keywords:** eccentric exercise, inflammation, circadian rhythm, cytokines, metabolism

## Abstract

**Background:**

The eccentric exercise performed during the day, whether in the morning or evening, may affect hormonal fluctuations and immune function. Furthermore, athletes frequently incorporate eccentric exercises into their training regimens or competitive events at these times, yet the inflammatory responses linked to this type of exercise remain unclear.

**Aim:**

The aim of this study was to investigate the impact of day time (morning vs. evening) eccentric exercise on inflammatory cytokines, antioxidant enzymes, and hormonal responses in physically active men.

**Methods:**

Using a randomized crossover design, twelve active men engaged in a session of plyometric type eccentric exercise (i.e., depth jump, 15 sets of 10 repetitions) in the morning and evening sessions, with blood samples collected pre (T1), immediately post (T2) and 1-h post-exercise (T3), to assess alterations in inflammatory cytokines (interleukin-6 [IL-6], IL-10, IL-1ra, and tumor necrosis factor alpha [TNF-α]), antioxidant enzymes (catalase [CAT], superoxide dismutase [SOD]), and glutathione peroxidase [GPx]), and hormonal changes (testosterone and cortisol).

**Results:**

Both the morning and evening eccentric exercise sessions indicated elevations in the cytokines with peak values at T2 (p=0.001), and enhancements of CAT and SOD with peaking values at T3 (p=0.001). While in the GPx, both the groups indicated their peaking elevations at the T2 (p=0.001). Morning group exhibited greater testosterone and cortisol concentrations than the evening group (p < 0.05), but these concentrations remained unchanged after the eccentric exercise session. A significant group × time interaction was observed in IL-6 (p=0.014), IL-10 (p=0.039), IL-1ra (p=0.001), TNF-α (p=0.021), CAT (p=0.046), SOD (p=0.001), GPx (p=0.016), testosterone (p=0.002) and cortisol (p=0.001), revealing greater changes for the evening compared to the morning exercise session.

**Conclusions:**

Variations in inflammatory cytokines, antioxidant enzymes, and hormonal responses to eccentric exercise in physically active young male are influenced by the time of day, with more pronounced responses observed in the evening than in the morning.

## 1 Introduction

Strength and conditioning coaches generally design different training programs in the specific phases of the annual training plan, aiming to enhance performance adaptations (1). In this context, they employ conditioning and sport-specific training sessions throughout the day (i.e., conditioning training in the morning and sports practice in the evening) (2). Athletes may demonstrate different responses to exercise training depending on the specific time of day at which each training program is implemented (3). The variations in physiological responses throughout the day are referred to as “circadian rhythm,” which influences the physical performance of athletes (4). In fact, the circadian system plays a crucial role in regulating various physiological functions, encompassing the endocrine, nervous, and immune systems (5, 6). Previous research has indicated that inflammatory cytokines, such as TNF-α and IL-6, display diurnal variations in human subjects (7). Furthermore, elevated levels of IL-6 have been associated with enhanced substrate metabolism (8) and antioxidant enzymes (9). Additionally, plyometric exercise (PE) which is recognized as an effective method for enhancing the physical performance of athletes in the specific phase of an annual training plan (10) is associated with an increase in muscle damage, inflammation, and cytokine responses (11, 12); however, the responses differ based on whether the exercise occurs in the morning or evening.

In fact, the eccentric actions involved in PE sessions may lead to microtrauma in muscle fibers (13), resulting in elevated levels of cytokines such as IL-6, IL-10, IL-1ra, and TNF-α (14). Furthermore, the inclusion of PE is correlated with an increase in antioxidant enzymes, including catalase (CAT), superoxide dismutase (SOD), and glutathione peroxidase (GPx) (15). Notably, both catabolic (i.e., cortisol) and anabolic (i.e., testosterone) hormones significantly influence the inflammatory responses to PE, with evidence suggesting that elevated cortisol levels are associated with increased muscle damage and IL-6 following PE (11). It is well-established that hormones play a significant role in either stimulating or inhibiting the secretion of cytokines (16), and consequently, these physiological responses to acute PE may vary significantly depending on the time of day it is performed.

Moreover, the daytime eccentric exercise, whether conducted in the morning or evening, may influence hormonal variations (17) and immune function (18) and this point of view is essential when designing a training plan. Research has established the relationship between circadian rhythm and exercise metabolism (19), highlighting its significant influence on immune function throughout the day. However, there is a lack of studies examining the impact of specific times of day on cytokine responses during eccentric exercise in humans. Additionally, athletes often engage in eccentric exercises in the morning or evening as part of their training programs and or competitive events, yet the inflammatory responses associated with this form of exercise remain unknown. Therefore, the aim of this study was to examine the effects of specific daytime (morning vs. evening) eccentric exercise on inflammatory cytokines, antioxidant and hormonal responses in physically active men.

## 2 Materials and methods

### 2.1 Registration and ethics approval

Participants were given detailed information about the possible risks and discomforts related to the study and also were required to sign informed consent statements. As the study protocol incorporated participants identified as ‘physical active’ and in accordance with the ethics committee of Shandong Women’s University, this study did not enter clinical trials that require registration and it should be approved (20240411364AA) by the Ethics and Medical Committees of the University, as well as conform to the latest version of the Declaration of Helsinki regarding human subjects.

### 2.2 Sample size estimation

The optimal sample size for this study was determined using G*Power software (Version 3.1.9.2, University of Kiel, Germany). The primary outcome variable of interest was the change in IL-6, which was analyzed through ANOVA. The analysis utilized an effect size of 0.23, a statistical power of 0.80, and maintained a type I error rate of 0.05, as highlighted in a previous study by Chatzinikolaou et al. (11), that investigated the effects of PE on inflammatory responses in physically active men. This calculation ensured a greater than 80% probability of detecting the anticipated effect size, indicating that ten participants were necessary to effectively analyze the observed differences in post-eccentric exercise responses across varying times of day. In order to reduce the risk of participant dropout while collecting data, the sample size was subsequently increased to twelve participants.

### 2.3 Participants

In this study, twelve physically active young males (age=26.2 ± 4.4 years, height=178.6 ± 4.5 cm, and body mass=79.5 ± 4.1 kg) with similar daily habits and fitness levels [Tier 1, (20)] volunteered to the participant. All participants had prior experience in PE but had not engaged in such training for at least three months before the study began. Those who had sustained injuries to their lower body within the three months prior to their participation, or who had any medical or orthopedic issues that could influence their involvement or performance, were excluded from the study. The participants continued their regular nutritional and lifestyle practices, avoided engaging in vigorous exercise, and did not use any anti-inflammatory medications for seven days before and during the study.

### 2.4 Participants’ chronotype

The Morningness-Eveningness Questionnaire developed by Horne and Östberg was employed to assess the chronotype of participants through a self-administered survey comprising 19 items (21). This instrument evaluates whether an individual identifies as a morning or evening based on their sleep/wake habits and preferred times for physical and mental activities. According to Horne and Östberg’s classification system (21), scores from 16 to 41 are categorized as evening types, scores from 42 to 58 as intermediate types, and scores from 59 to 86 as morning types. Consequently, we selected physically active men with an intermediate chronotype to mitigate the influence of morningness or eveningness on the study’s outcomes.

### 2.5 Experimental design

**Figure 1:**
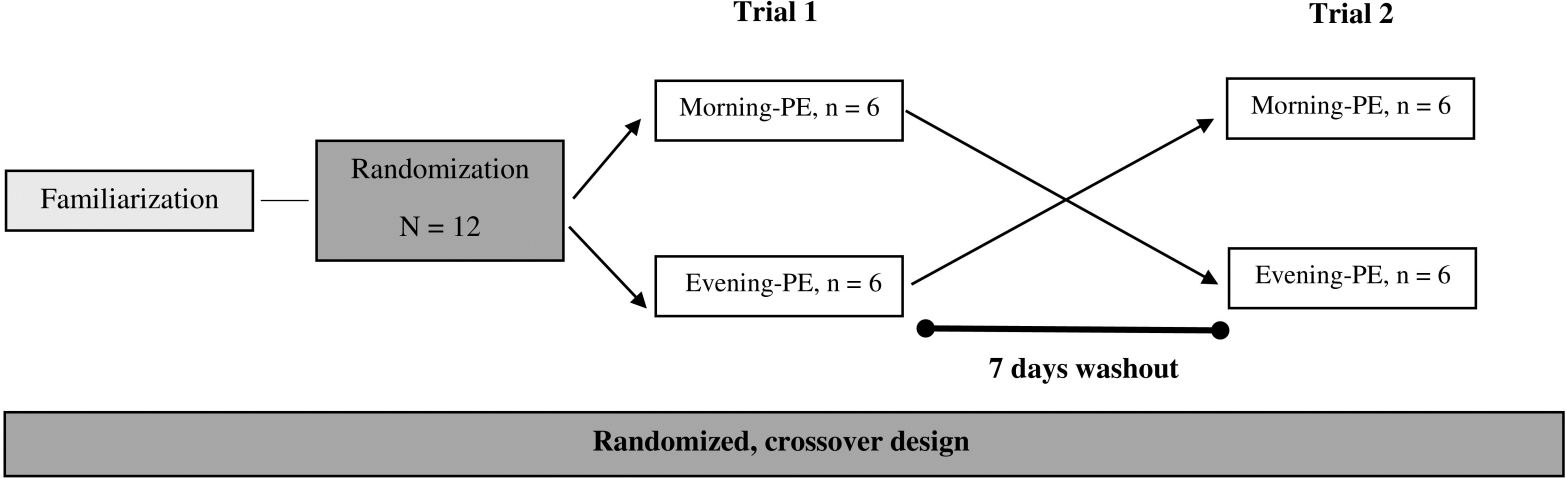
Cross-over design.

This research utilized a randomized crossover design to examine the effects of daytime PE on cytokine, antioxidant, and hormonal responses in male participants, following the methodology established by Kim et al. (19). A total of twelve physically active males were randomly assigned, using block randomization, to perform PE sessions either in the morning (n = 6) or in the evening (n = 6) (**Figure 1**). Each participant completed two experimental trials—one in the morning (09:00) and one in the evening (17:00)—with a 7-day washout period between trials to minimize residual effects (19). The order of morning and evening sessions was counterbalanced across participants to control for potential order effects. During the initial familiarization visit, the characteristics of the participants (height with a wall-mounted stadiometer [ ± 0.5 cm, Bodymeter, Germany] and body mass with a digital scale [ ± 0.1 kg, Ironman Body Composition Monitor, USA]) were assessed, and they received guidance on the correct techniques for executing PE (i.e., depth jumps). One week following this visit, the participants returned to the laboratory to engage in the PE session, during which blood samples were collected at three intervals: before (T1), immediately after (T2), and one-hour post-exercise (T3) to assess variations in cytokine, antioxidant, and hormonal levels. Participants were required to achieve a minimum of eight hours of sleep and were instructed to refrain from any vigorous physical activity for at least 72 hours leading up to the experimental day. These protocols were maintained for each participant during the study and were confirmed through personal interviews.

To ensure uniformity in the dietary intake among participants, three days of self-reported food records were collected prior to the start of the study. These records were subsequently evaluated using Nutritionist PROTM analysis software to calculate the total calorie intake (2850 ± 325 Kcal), protein (107 ± 13 g), carbohydrate (427 ± 29 g), fat (79 ± 11 g), vitamin C (8.3 ± 1.5 mg), and vitamin E (66 ± 12 mg). Participants received clearly instructions to continue their regular dietary habits and to avoid any supplementary intake throughout the duration of the study. All participants consumed their final pre-session meal at least 2 hours before exercise, with this interval kept consistent across morning and evening sessions.

### 2.7 PE intervention

Following a 15-minute warm-up that included 5 minutes of running and 10 minutes of stretching and ballistic movements (22, 23), the participants engaged in an eccentric exercise regimen comprising 15 sets of 10 repetitions of depth jumps from a 45-cm box, as recommended by Chu (10) and Arazi et al. (24). A one-minute rest interval was implemented between sets to facilitate adequate recovery. This protocol has been effectively utilized in prior research to elicit muscle damage and inflammation (25, 26). Participants were instructed to execute the eccentric exercise by standing on the 45-cm box, leading with one foot as they descended and landed with both feet on a firm surface (i.e., a wooden volleyball court). Upon making firm contact, they were directed to jump off the ground as quickly and as high as possible. Participants were instructed to land softly with approximately 90° knee flexion, minimize ground contact time, and maintain neutral spine alignment during landings. Throughout the session, verbal encouragement was consistently provided to motivate participants to exert their maximum effort during each trial, with a specialized strength and conditioning coach closely supervising the exercise protocols (27). The morning and evening PE sessions were conducted at 9:00 AM and 5:00 PM, respectively, with a temperature range of 27-29°C and humidity of 40-45%.

### 2.8 Blood sampling and analysis

Blood samples were collected prior to exercise (T1) after a 15-minute period of seated rest. Subsequent samples were taken immediately after exercise (i.e., 1-minute) (T2) (28) and one-hour post-exercise (T3), following a seated rest of one minute and one hour, respectively. A 15 cc sample was drawn from the antecubital vein using plain evacuated test tubes. The blood was allowed to clot at room temperature for 30 minutes before being centrifuged at 1500 × g for 10 minutes. The serum was then separated and stored in multiple aliquots at -20°C for future analysis. The levels of IL-6, IL-10, IL-1ra, and TNF-α were quantified using a commercially available ELISA kit (R&D Systems Inc, UK) and analyzed with a spectrophotometric plate reader (Dynex Technologies 268 Inc, USA). Serum testosterone and cortisol levels were assessed through the radioimmunoassay method, adhering to standardized procedures with available kits (Monobind, Inc., Lake Forest, CA, USA). Additionally, antioxidant enzymes, including CAT, SOD, and GPx, were measured using an ELISA kit (Cayman Chemical Company, Ann Arbor, MI, USA). The coefficient of variation for all blood measurements remained below 6%.

### 2.9 Statistical analysis

Prior to conducting statistical comparisons, all data were evaluated for normal distribution using the Shapiro-Wilk test. The results are expressed as mean ± standard deviation (SD). A two-way repeated-measures ANOVA, specifically a 2 (group; morning vs. evening) x 3 (time; T1, T2 and T3) design, was performed using the SPSS statistical software package (version 21.0 for Windows, SPSS Inc., Chicago, IL, United States) to analyze the data. In cases where a significant F value was observed, a Bonferroni *post hoc* test was employed to detect differences in the measurements. Furthermore, absolute values were presented to illustrate the main data of the measured variables and to make the primary findings of the study clearly visible, while percent changes from baseline (T1) were calculated as: [(value at time point – baseline value)/baseline value] × 100, and percent change score differences between the pre-measurements and all subsequent time points were calculated and analyzed using a t-test to assess the differences in variables between morning and evening PE sessions. In addition, the effect size (ES) was calculated with a 95% confidence interval (CI) to evaluate the magnitude of changes between morning and evening groups. The significance level was set at 0.05.

## 3 Results

All participants attended every session, demonstrating complete adherence, which resulted in a success rate of 100%. At baseline, there were no significant differences between the morning and evening groups in the measured variables, with the exception that the morning group exhibited higher baseline levels of testosterone and cortisol compared to the evening group (p < 0.05).

**Figure 2:**
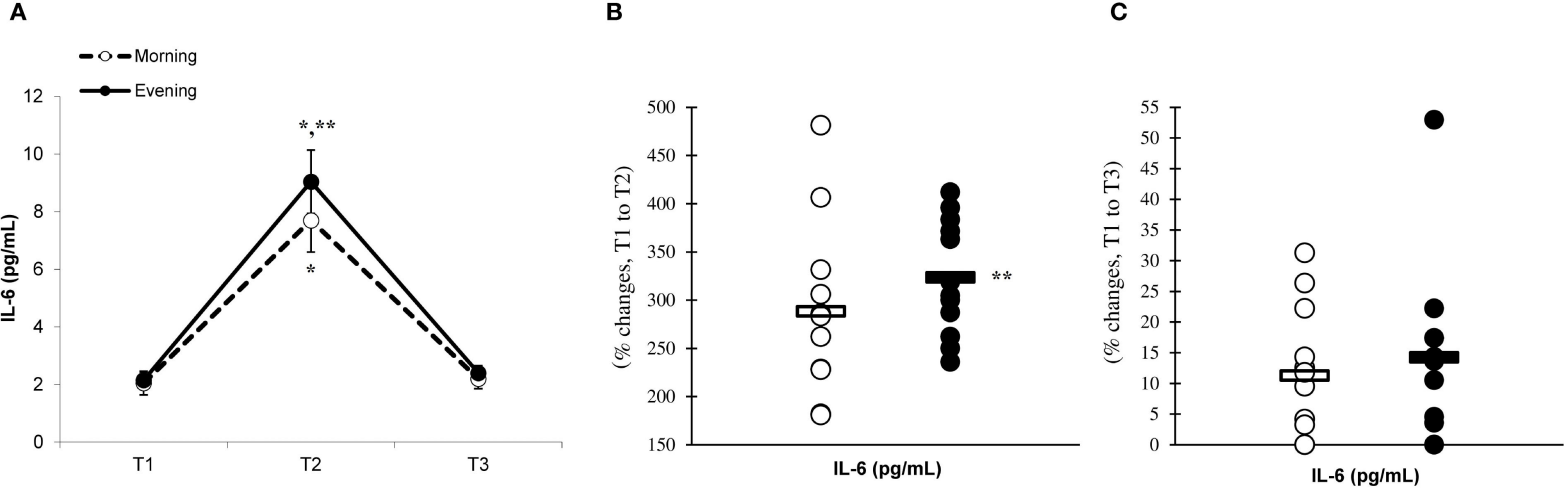
Time-dependent variations in absolute IL-6 **(A)** and the magnitude of change from T1 to T2 **(B)** and from T1 to T3 **(C)** (mean ± SD). *Significantly different from T1 (p < 0.05); **Significantly different from the morning group (p < 0.05).

**Figure 3:**
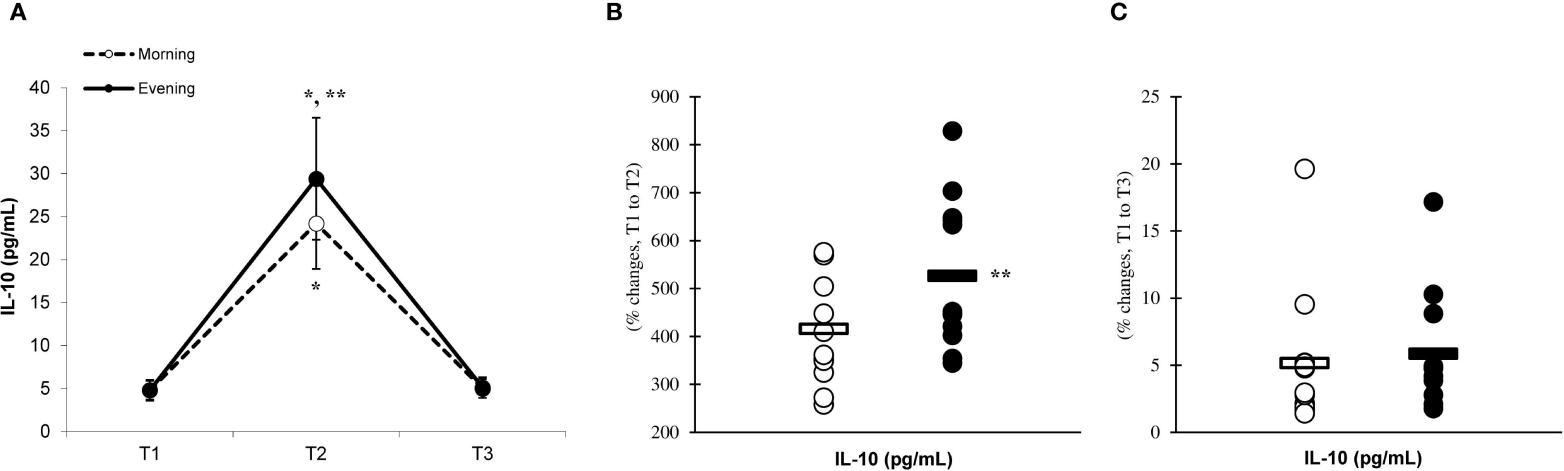
Time-dependent variations in absolute IL-10 **(A)** and the magnitude of change from T1 to T2 **(B)** and from T1 to T3 **(C)** (mean ± SD). *Significantly different from T1 (p < 0.05); **Significantly different from the morning group (p < 0.05).

**Figure 4:**
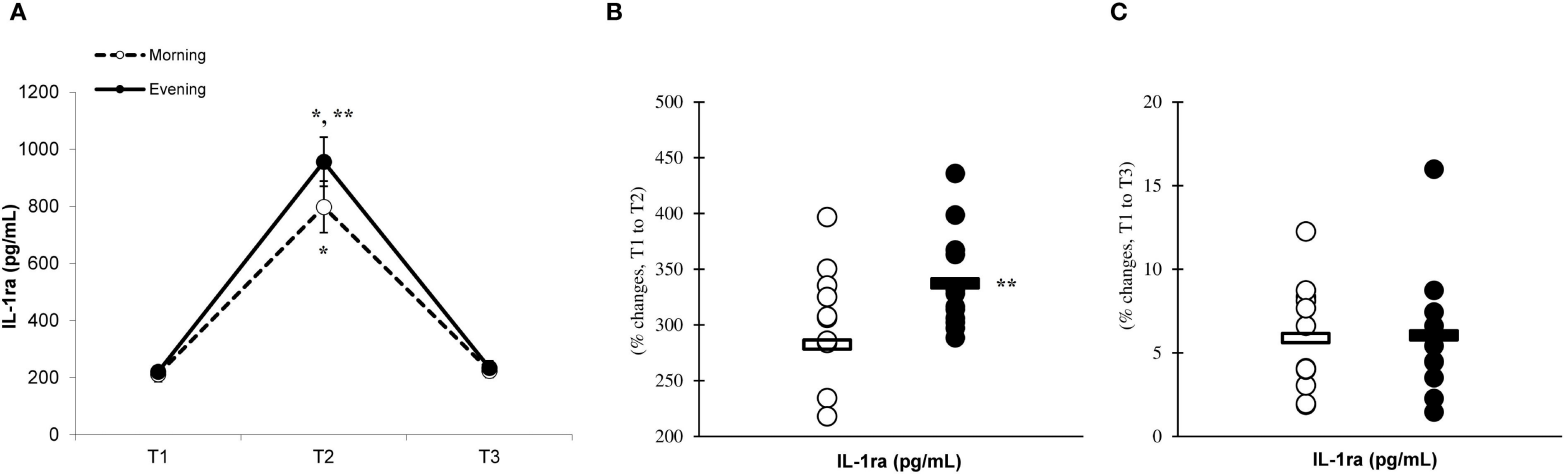
Time-dependent variations in absolute IL-1ra **(A)** and the magnitude of change from T1 to T2 **(B)** and from T1 to T3 **(C)** (mean ± SD). *Significantly different from T1 (p < 0.05); **Significantly different from the morning group (p < 0.05).

**Figure 5:**
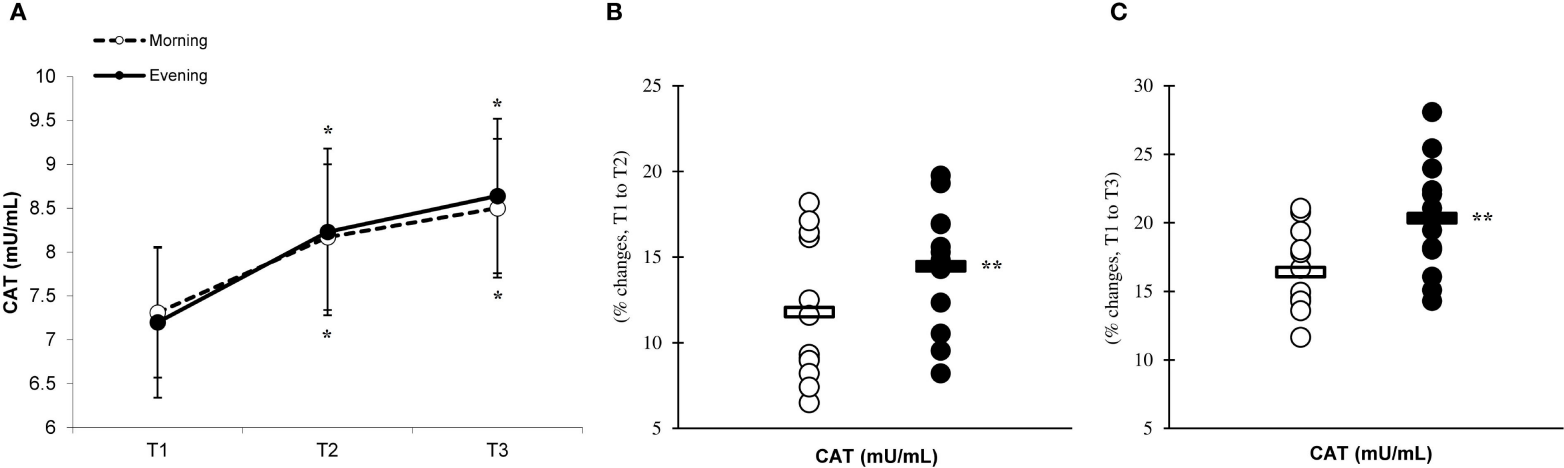
Time-dependent variations in absolute TNF-α **(A)** and the magnitude of change from T1 to T2 **(B)** and from T1 to T3 **(C)** (mean ± SD). *Significantly different from T1 (p < 0.05); **Significantly different from the morning group (p < 0.05).

There was a significant main effect of time (p=0.001) in IL-6 (**Figure 2A**), IL-10 (**Figure 3A**), IL-1ra (**Figure 4A**), and TNF-α (**Figure 5A**), demonstrating that peak values were attained at T2 for both the morning and evening groups, followed by a return to baseline levels at T3 for both groups. A significant group × time interaction was observed in IL-6 (F=6.809, p=0.014), IL-10 (F=4.790, p=0.039), IL-1ra (F=16.529, p=0.001), and TNF-α (F=6.175, p=0.021). Follow-up analysis showed more changes for the evening group in the IL-6 (323.6% ± 60.1% vs. 288.3% ± 87.3%, p=0.001, ES=1.18, 95% CI=0.31 to 2.04, **Figure 2B**), IL-10 (526.3% ± 156.5% vs. 415.8% ± 115.6%, p=0.001, ES=0.80, 95% CI=-0.03 to 1.63, **Figure 3B**), IL-1ra (337.2% ± 45.1% vs. 228.4% ± 70.3%, p=0.001, ES=1.73, 95% CI=0.79 to 2.67, **Figure 4B**), and TNF-α (195.3% ± 65.7% vs. 164.6% ± 62.1%, p=0.001, ES=1.31, 95% CI=0.43 to 2.19, **Figure 5B**) than the morning group at the T2, while no significant differences were noted between the groups at the T3 (p > 0.05) (**Figures 2**–**4**, **5C**).

**Figure 6:**
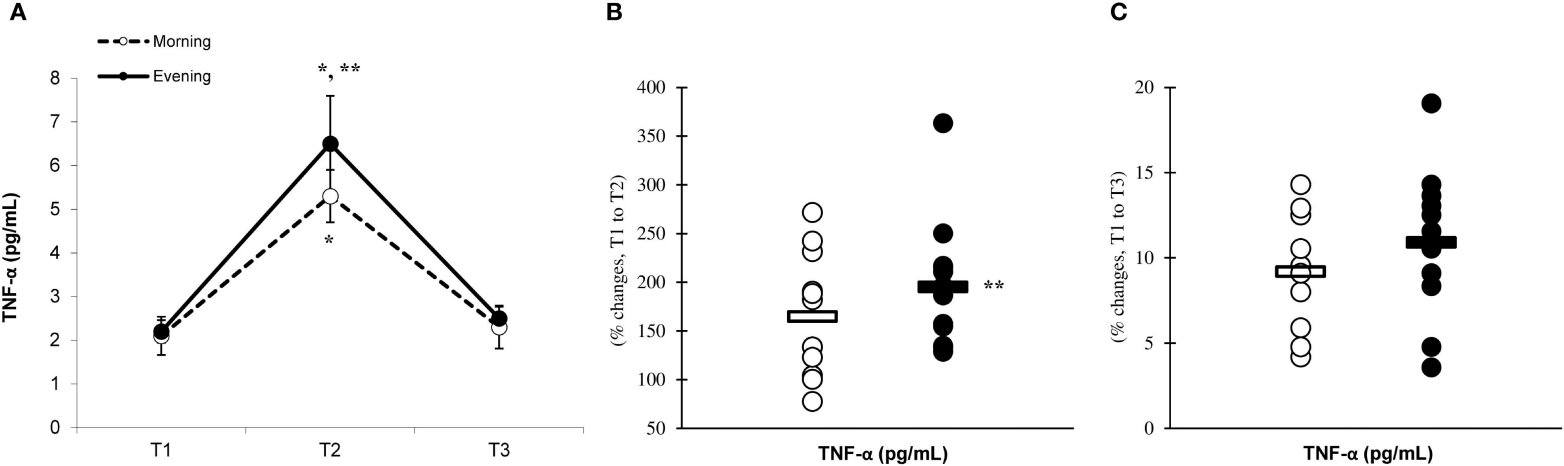
Time-dependent variations in absolute CAT **(A)** and the magnitude of change from T1 to T2 **(B)** and from T1 to T3 **(C)** (mean ± SD). *Significantly different from T1 (p < 0.05); **Significantly different from the morning group (p < 0.05).

**Figure 7:**
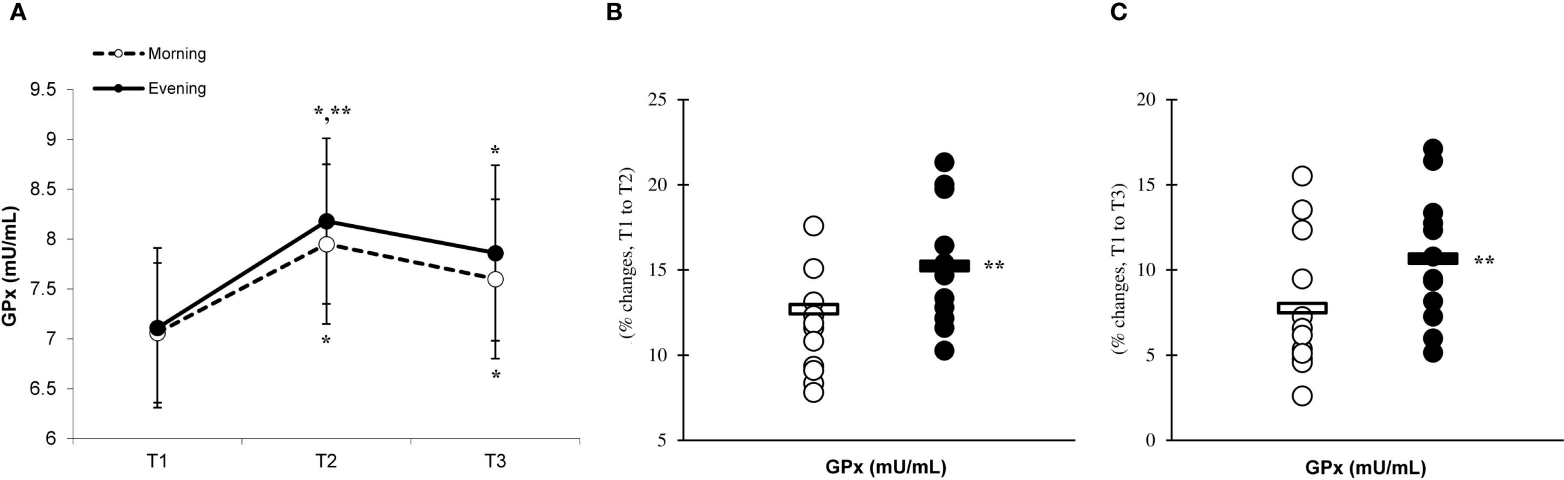
Time-dependent variations in absolute SOD **(A)** and the magnitude of change from T1 to T2 **(B)** and from T1 to T3 **(C)** (mean ± SD). *Significantly different from T1 (p < 0.05); **Significantly different from the morning group (p < 0.05).

**Figure 8:**
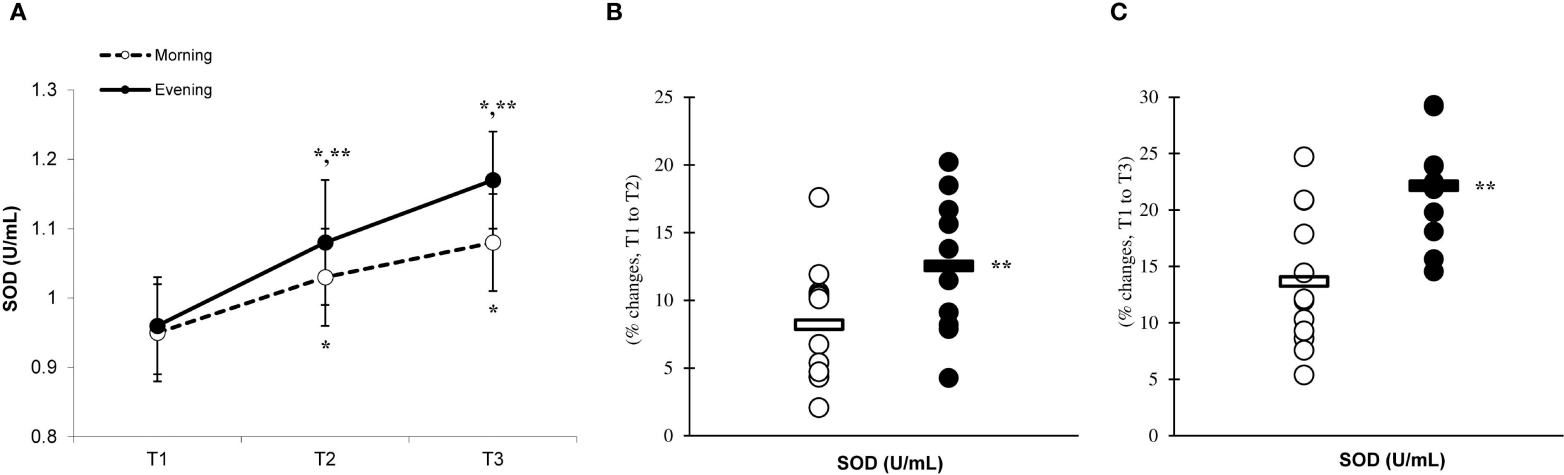
Time-dependent variations in absolute GPx **(A)** and the magnitude of change from T1 to T2 **(B)** and from T1 to T3 **(C)** (mean ± SD). *Significantly different from T1 (p < 0.05); **Significantly different from the morning group (p < 0.05).

There was a significant main effect of time (p=0.001) in CAT (**Figure 6A**), SOD (**Figure 7A**), and GPx (**Figure 8A**), demonstrating progressively increases in the CAT and SOD from T1 to T3, with peak values attained at T3 for both the morning and evening groups. Meanwhile, in the GPx levels, both the morning and evening groups showed peak values at T2 while maintaining their elevation at T3 from the corresponding T1. A significant group × time interaction was observed in CAT (F=3.651, p=0.046), SOD (F=10.223, p=0.001), and GPx (F=9.339, p=0.016). Follow-up analysis showed more changes for the evening group in the CAT (14.4% ± 3.6% vs. 11.7% ± 4.1%, p=0.022, ES=0.06, 95% CI=-0.74 to 0.87, **Figure 6B**), SOD (12.5% ± 5.6% vs. 8.2% ± 4.3%, p=0.001, ES=0.60, 95% CI=-0.22 to 1.42, **Figure 7B**), and GPx (15.2% ± 3.5% vs. 12.6% ± 4.8%, p=0.028, ES=0.27, 95% CI=-0.53 to 1.08, **Figure 8B**) than the morning group at the T2. In addition, significant differences were noted between the evening and morning groups for the magnitude of changes in the CAT (20.3% ± 4.2% vs. 16.4% ± 3.1%, p=0.011, ES=0.16, -0.64 to 0.96, **Figure 6C**), SOD (22.1% ± 6.0% vs. 13.6% ± 6.1%, p=0.001, ES=1.24, 95% CI=0.37 to 2.12, **Figure 7C**), and GPx (10.6% ± 3.8% vs. 7.7% ± 4.1%, p=0.032, ES=0.30, -0.51 to 1.10, **Figure 8C**) at the T3.

**Figure 9:**
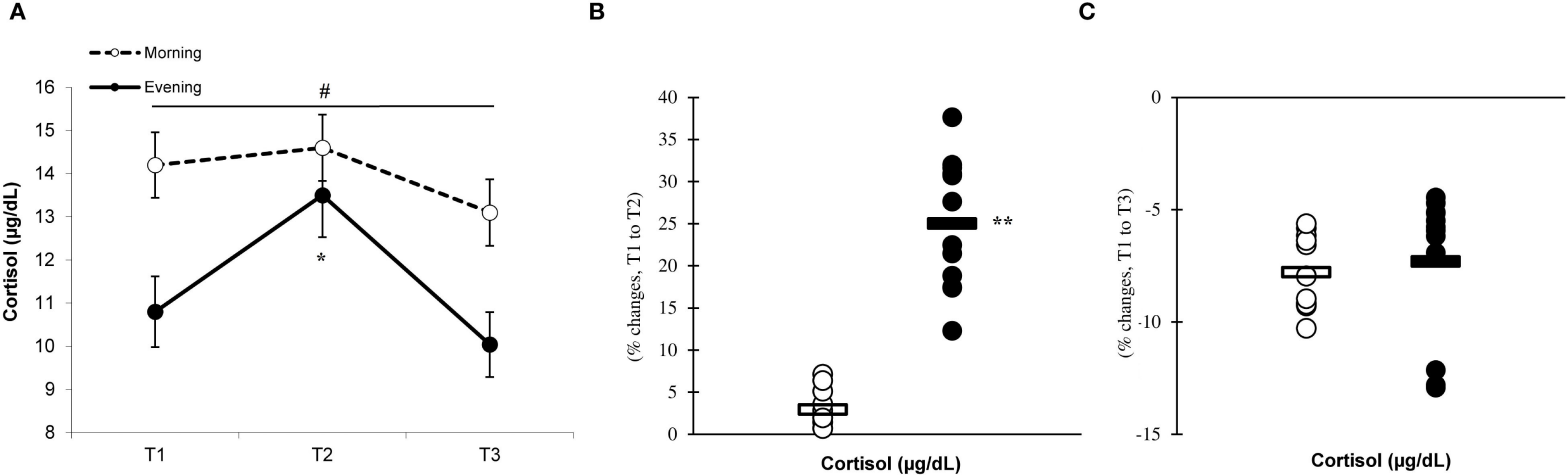
Time-dependent variations in absolute testosterone **(A)** and the magnitude of change from T1 to T2 **(B)** and from T1 to T3 **(C)** (mean ± SD). *Significantly different from T1 (p < 0.05); **Significantly different from the morning group (p < 0.05). ^**#**^Indicates significant differences at all T1, T2, and T3 time points between the morning and evening groups (p < 0.05).

**Figure 10:**
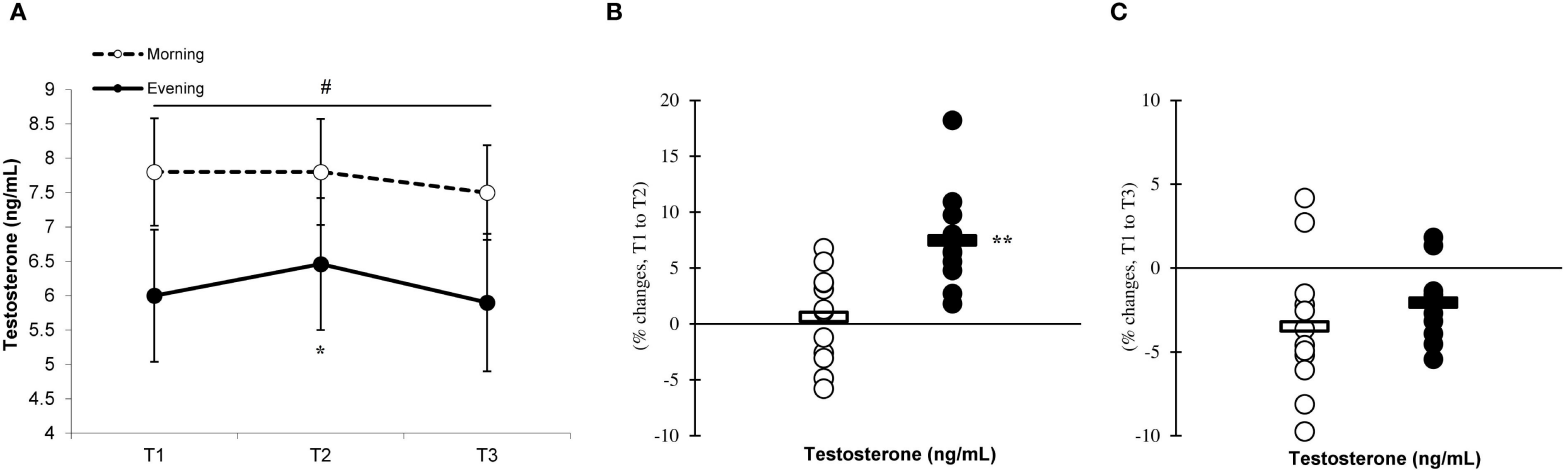
Time-dependent variations in absolute cortisol **(A)** and the magnitude of change from T1 to T2 **(B)** and from T1 to T3 **(C)** (mean ± SD). *Significantly different from T1 (p < 0.05); **Significantly different from the morning group (p < 0.05). ^**#**^Indicates significant differences at all T1, T2, and T3 time points between the morning and evening groups (p < 0.05).

Significant differences (p < 0.05) between the groups were observed at T1, T2, and T3, with the morning group showing greater testosterone and cortisol concentrations than the evening group. The analysis revealed significant effects of time (p=0.001) and a group × time interaction for testosterone (F=7.883, p=0.002) (**Figure 9A**) and cortisol (F=6.778, p=0.001) (**Figure 10A**) levels, indicating that the evening group experienced more pronounced changes at T2 compared to the morning group (testosterone, 7.4% ± 4.2% vs. 0.6% ± 4.1%, p=0.001; cortisol, 25.1% ± 7.7% vs. 2.9% ± 2.1%, p=0.001) (**Figures 9**, **10B**). Both groups showed a decline in testosterone and cortisol levels at T3 in relation to T1 (**Figures 9C**, **10C**), although these variations did not reach statistical significance (p > 0.05).

## 4 Discussion

This research sought to investigate the impact of daytime eccentric exercise on the alterations in inflammatory cytokines, antioxidant enzymes, and hormonal responses in humans. This was one of the first examinations in this area to clarify the influence of time of day during eccentric exercise on cytokine, antioxidant and hormonal changes in physically active men. Our results indicated that a session of eccentric exercise led to an increase in both inflammatory cytokines and antioxidant enzymes. Notably, these changes were more pronounced in the evening group compared to the morning group, which exhibited greater responses in cytokines and antioxidant enzymes during the evening. Regarding hormonal responses, the morning group displayed greater testosterone and cortisol concentrations (i.e., at T1, T2, and T3), while only the evening group experienced increases in testosterone and cortisol following the eccentric exercise.

We found that a session of high-intensity PE led to significant increases in IL-6, IL-10, IL-1ra, and TNF-α at T2, with these levels returning to baseline at T3. These results are consistent with prior studies that documented elevations in inflammatory cytokines following eccentric exercise in both men and women (26, 29, 30); however, others reported contradictory findings (31). The discrepancies in these results may be attributed to various factors, including the type of eccentric exercise (such as resistance and endurance versus plyometric), the intensity of the exercise (measured as a percentage of maximal effort versus all-out maximal effort), and the extent of muscle mass engaged during the activity (for instance, targeting a specific muscle group like the quadriceps versus involving the entire leg during jumping) (32).

The observed increase in IL-6 expression following PE is associated with a rise in neutrophils and macrophages (11), which may significantly contribute to the elevation of pro-inflammatory cytokines in the plasma and the potential for subsequent tissue damage and upregulation of IL-10 and IL-1ra levels in the bloodstream (29). The enhanced secretion of IL-10 during intense eccentric exercise may facilitate a shift in the immune response towards an anti-inflammatory pathway by inhibiting the production of pro-inflammatory cytokines such as TNF-α (14, 18). Typically, the sequence of cytokine release in response to eccentric exercise begins with an initial increase in serum IL-6 levels, followed by subsequent rises in IL-1ra, IL-10, and TNF-α concentrations (18). Additionally, elevated cortisol levels may significantly contribute to increased cytokine production in the bloodstream, resulting in more pronounced responses in the evening group compared to the morning group (19). Specifically, the evening group exhibited heightened levels of inflammatory cytokines at T2 when contrasted with the morning group. These results indicate a greater cytokine response during the evening, corroborating findings by Kim et al. (19), who noted higher IL-6 and cortisol levels in the evening group relative to the morning group. Thus, it can be concluded that the cytokine responses to eccentric exercise are influenced by the time of day, with evening exercise potentially leading to more substantial increases in cortisol and adrenaline, thereby enhancing cytokine levels more than morning exercise. Evening exercise may amplify inflammatory responses through greater activation of the NF-KB signaling pathway and increased mitochondrial ROS production (8). Because cortisol follows a pronounced circadian rhythm, peaking in the morning and declining toward the evening, lower evening concentrations provide less anti-inflammatory buffering compared with morning conditions (16). This reduced hormonal suppression may permit enhanced oxidative signaling, thereby facilitating NF-KB–mediated transcription of pro-inflammatory cytokines (8, 19). Consequently, the interplay between circadian cortisol variation, mitochondrial ROS generation, and NF-KB activation may create a more pro-inflammatory milieu during evening exercise, potentially explaining the more pronounced cytokine responses observed compared with morning sessions (16, 19).

Our investigation revealed that engaging in high-intensity PE significantly elevated the levels of CAT, SOD, and GPx. These findings are in agreement with previous research that demonstrated increases in antioxidant enzymes following eccentric exercise among both genders (33, 34). The results of the current investigation revealed that the recovery period following eccentric exercise led to an increase in the levels of CAT, SOD, and GPx. These findings indicate that the extent of these responses correlates with the degree of inflammation experienced after exercise (35, 36). Indeed, previous studies proposed that the degree of enhancement in the antioxidant defense system is contingent upon the level of inflammation, with the activation of CAT, SOD, and GPx being crucial for mitigating the effects of free radicals and inflammation subsequent to exercise (37–40).

Our research indicates that the responses of CAT, SOD, and GPx to exercise are more pronounced in the evening group than in the morning group, suggesting a heightened involvement of antioxidant enzymes during evening workouts. This phenomenon seems to be linked to the cytokine responses elicited by eccentric exercise during plyometric activity (11). Specifically, the evening group demonstrated enhanced inflammatory responses, which could have facilitated more adaptive responses, resulting in the upregulation of antioxidant enzymes due to eccentric exercise conducted in the evening (19).

The production of anabolic and catabolic hormones, including testosterone and cortisol, is determined by the intensity of the exercise performed. These hormones also exhibit diurnal variations, with higher concentrations in the morning than in the evening. Our findings demonstrated that the morning group had significantly greater concentrations of testosterone and cortisol at T1, T2, and T3 compared to the evening group. Moreover, the evening group displayed more pronounced changes in testosterone and cortisol levels at T2 in response to eccentric exercise when compared to the morning group. These findings may provide insight into how evening eccentric exercise influences the responses of testosterone and cortisol (11, 17). Furthermore, the morning group exhibited no alterations in testosterone and cortisol levels following exercise, which may be attributed to their elevated baseline levels of these hormones. These results align with prior research by Kim et al. (19), who observed that engaging in exhaustive endurance exercise in the evening elicited greater cortisol responses compared to morning session. Additionally, their study indicated that the morning group did not experience any increases in cortisol levels post-exercise (19), corroborating our findings. Overall, the enhanced hormonal responses associated with evening exercise compared to the morning sessions may be influenced by higher levels of adrenaline and noradrenaline, as well as changes in the catabolic state related to the time of day during eccentric exercise (41). The larger relative changes observed in hormonal responses during evening exercise may, in part, reflect the influence of lower baseline concentrations at that time of day. From a physiological perspective, when baseline hormone levels such as testosterone and cortisol are reduced—as typically observed in the evening—the same absolute post-exercise increase will constitute a proportionally greater relative change (19). This baseline-dependent amplification may also interact with circadian variations in hormone sensitivity and receptor expression, resulting in more pronounced reactive responses despite lower absolute levels. Thus, the evening profile may represent not only a different hormonal starting point but also a distinct regulatory environment that magnifies the relative magnitude of exercise-induced hormonal shifts. From a practical standpoint, these findings suggest that scheduling eccentric exercise in the evening may elicit stronger inflammatory and antioxidant responses, potentially influencing recovery strategies, training load management, and competition timing. Coaches and practitioners should account for time-of-day variations when developing training programs for physically active men, as evidence suggests that circadian timing can influence key physiological responses— including inflammatory cytokine levels, antioxidant activity, and hormonal fluctuations—which may play a critical role in determining the adaptive benefits achieved through training.

In light of these results, several limitations must be acknowledged. While the crossover design effectively reduced inter-individual variability, the sample comprised only young, physically active males. Consequently, the generalizability of the findings to other populations—such as females, older individuals, or elite athletes—should be approached with caution. This study did not assess additional metabolic or muscle damage indicators, nor did it evaluate other endocrine markers such as growth hormone (GH), which limits the ability to provide a mechanistic interpretation of the results. This limitation also prevents correlating observed cytokine surges with direct measures of muscle damage (e.g., creatine kinase) or subsequent repair processes, restricting mechanistic interpretation. Further research is required to determine whether these findings can be applied to athletic populations, including basketball and handball players, as well as across various age groups. Lastly, it would have been advantageous to include a range of muscle functional tests after plyometric exercises. Future studies should consider these aspects to strengthen our conclusions.

## 5 Conclusions

The findings of this research suggest that engaging in a session of lower-body eccentric exercise can lead to an elevation in inflammatory cytokines and antioxidant enzymes, along with hormonal alterations specifically noted in the evening sessions among physically active males. Furthermore, it was noted that the responses of cytokines and antioxidants were more pronounced in the evening session. Conversely, the evening group exhibited elevated responses in testosterone and cortisol levels when compared to the morning group, which demonstrated higher baseline levels. It is important to note that variations in inflammatory cytokines, antioxidant enzymes, and hormonal responses to eccentric exercise are influenced by the time of day, with more pronounced responses observed during evening sessions compared with morning sessions in physically active young men. Coaches and practitioners should account for time-of-day variations when developing training programs for physically active men, as circadian rhythms can influence physiological responses which may, in turn, shape the adaptive benefits of training.

## Data Availability

The raw data supporting the conclusions of this article will be made available by the authors, without undue reservation.
